# Characterization and Prevalence of a New Porcine Calicivirus in Swine, United States

**DOI:** 10.3201/eid1706.101756

**Published:** 2011-06

**Authors:** Qiuhong Wang, Kelly Scheuer, Zhenwen Zhang, Wondwossen A. Gebreyes, Bayleyegn Z. Molla, Armando E. Hoet, Linda J. Saif

**Affiliations:** Author affiliations: The Ohio State University, Wooster, Ohio, USA (Q. Wang, K. Scheuer, Z. Zhang, L.J. Saif);; The Ohio State University, Columbus, Ohio, USA (W.A. Gebreyes, B.Z. Molla, A.E. Hoet)

**Keywords:** calicivirus, St-Valerien-like virus, porcine, prevalence, viruses, swine, United States, dispatch

## Abstract

Real-time reverse transcription PCR revealed that new St-Valerien–like porcine caliciviruses are prevalent (2.6%–80%; 23.8% overall) in finisher pigs in North Carolina. One strain, NC-WGP93C, shares 89.3%–89.7% genomic nucleotide identity with Canadian strains. Whether these viruses cause disease in pigs or humans or are of food safety concern requires further investigation.

Viruses in the family *Caliciviridae* are nonenveloped, polyadenylated, single-stranded, positive-sense RNA viruses (*1*). They have been classified into 5 genera (*Norovirus*, *Sapovirus*, *Vesivirus*, *Lagovirus,* and *Nebovirus*) since 2009 (www.ictvonline.org). Later, the nonhuman primate Tulane virus (*2*) and the porcine St-Valerien–like viruses (*3*) were characterized as potential new genera in the *Caliciviridae* family.

## The Study

**Figure 1:**
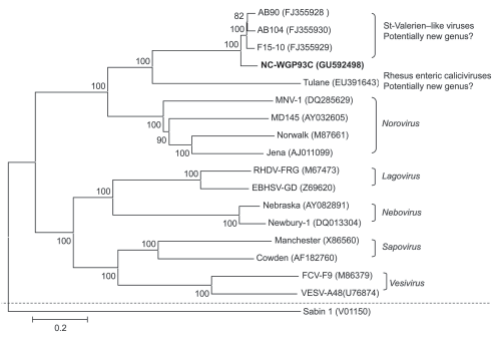
Neighbor-joining phylogenetic tree of caliciviruses based on the complete genomes (nucleotide). The newly identified St-Valerien–like virus NC-WGP93C strain is in **boldface**. The GenBank accession number of each strain is within parentheses. Bootstrap values are shown near branches. Human *Poliovirus* Sabin 1 was an outgroup control. Scale bar indicates nucleotide substitutions per site.

Recently, we identified a St-Valerien–like virus, NC-WGP93C strain, from a healthy finisher pig in the United States by reverse transcription PCR (RT-PCR) with calicivirus universal primers p290/110 (*4**,**5*), followed by direct sequencing and nucleotide BLAST search (www.ncbi.nlm.nih.gov). We further sequenced the genome of NC-WGP93C strain by using primer walking, 3′ and 5′ rapid amplification of cDNA ends (RACE) methods (*3**,**6**,**7*). The NC-WGP93C strain was closely related genetically to the Canadian St-Valerien–like viruses, AB90, AB104, and F15–10 strains (*3*), sharing 89.3%–89.7% nt identity, without insertions or deletions, and similar genomic organization. Complete genomes of strains representing different *Caliciviridae* genera were selected for a phylogenetic tree (Figure 1). The NC-WGP93C strain grouped with the Canadian St-Valerien–like viruses to form a potentially new genus within the *Caliciviridae* family.

Next, we developed a real-time quantitative RT-PCR (RT-qPCR) for detection of St-Valerien–like viruses with primers (WGP93-polF1, 5′-TCTAAAGCGTGCACTCTGGGTCAT-3′; WGP93-polR1, 5′-ACCCTTTCTCCACCAGGAACTTCT-3′) and probe (WGP93-polP1, FAM-ACGAGTTTGTGGACTTCCTCTCGCA-BHQ) that targeted the RNA-dependent RNA polymerase (RdRp). The assay was performed by using the OneStep RT-PCR Kit (QIAGEN, Valencia, CA, USA) and a real-time thermocycler (RealPlex, Eppendorf, Germany). A plasmid DNA carrying the p290/110 amplicon of the NC-WGP93C strain was used to generate a standard curve. The detection limit was 10 genomic equivalents (GE) per 20-µL reaction (cycle threshold 37.71), corresponding to 4 × 10^4^ GE/g of fecal sample (cut-off cycle threshold 38.00). No other porcine enteric caliciviruses, including sapoviruses (GIII/Cowden, GVI/JJ681, GVII/LL26 strains) and noroviruses (GII.11/QW48, GII.18/QW101, and GII.19/QW170 strains) (*8**,**9*), were detected. This RT-qPCR is sensitive and specific for the detection of St-Valerien–like caliciviruses.

Using the above RT-qPCR, we performed a prevalence study of St-Valerien–like viruses. Pig fecal samples (n = 1,567) were collected during May–November 2009 from a study of *Salmonella* infections in apparently healthy finisher pigs from 3 different swine production systems (3 farms per system, and 4 barns per farm, except for farm RW [3 barns]) located in North Carolina (Table 1) (*10*). Each barn was treated with 1 of 3 biocides—Biosentry (Biosentry, Inc., Stone Mountain, GA, USA), Synergize (Preserve International, Reno, NV, USA), or VirkonS (Dupont Animal Health Solutions, Sudbury, UK)—or with pressurized water as control (*11*). One pig per pen was sampled at 26–28 weeks of age. Fecal samples were collected directly from the rectums of selected individual pigs (based on convenience); sterile gloves and cups were used to prevent contamination from the environment and between samples. In the laboratory, 4–5 individual samples from the same barn were pooled and stored (–20°C), resulting in 344 pooled fecal samples for the prevalence study. RNA was extracted from 10% (wt/vol) fecal suspensions by using the 5× MagMAX-96 Viral 1 Kit and the RNA extraction robot MagMax Express Magnetic Particle Processor (Applied Biosystems, Foster City, CA, USA). The relationship and differences in prevalence among the various biocide treatments (barns), farms, and production systems were assessed by χ^2^ and binomial proportion tests (SAS Institute, Inc., Cary, NC, USA). A p value of <0.05 was considered significant.

**Table 1:** Prevalence of St-Valerien–like viruses in finisher swine farms in North Carolina, USA, 2009*

System code	Farm code	Barn treatment	Sampling month	No. individual samples	No. pooled samples	Barn level, no. positive/total (%)	Farm level, no. positive/total (%)†
BC1	BH	Water	Aug	48	10	4/10 (40.0)	8/40 (20.0)^bc^
BC1	BH	BIO	Aug	48	10	2/10 (20.0)	
BC1	BH	SYN	Aug	48	10	0/10	
BC1	BH	VIR	Aug	48	10	2/10 (20.0)	
BC1	EW	Water	Jul	42	10	0/10	1/39 (2.6)^c^
BC1	EW	BIO	Jul	39	9	0/10	
BC1	EW	SYN	Jul	47	10	1/10 (10.0)	
BC1	EW	VIR	Jul	48	10	0/10	
BC1	WL	Water	May	47	10	10/10 (100.0)	32/40 (80)^a^
BC1	WL	BIO	May	47	10	10/10 (100.0)	
BC1	WL	SYN	May	46	10	2/10 (20.0)	
BC1	WL	VIR	May	48	10	10/10 (100.0)	
BC2	DC	Water	Nov	39	9	4/9 (44.4)	13/37 (35.1)^b^
BC2	DC	BIO	Nov	45	10	7/10 (70.0)	
BC2	DC	SYN	Nov	35	8	2/8 (25.0)	
BC2	DC	VIR	Nov	48	10	0/10	
BC2	FF	Water	Oct	43	10	1/10 (10.0)	3/40 (7.5)^c^
BC2	FF	BIO	Oct	48	10	1/10 (10.0)	
BC2	FF	SYN	Oct	40	10	0/10	
BC2	FF	VIR	Oct	48	10	1/10 (10.0)	
BC2	RW	BIO	May	45	10	0/10	2/30 (6.7)^c^
BC2	RW	SYN	May	48	10	2/10 (20.0)	
BC2	RW	VIR	May	46	10	0/10	
BC3	GO	Water	Nov	46	10	1/10 (10.0)	8/40 (20)^bc^
BC3	GO	BIO	Aug	45	10	1/10 (10.0)	
BC3	GO	SYN	Nov	46	10	4/10 (40.0)	
BC3	GO	VIR	Aug	46	10	2/10 (20.0)	
BC3	TE	Water	Jul	47	10	4/10 (40.0)	12/39 (30.8)^b^
BC3	TE	BIO	Jul	38	9	2/9 (22.2)	
BC3	TE	SYN	Jul	40	10	4/10 (40.0)	
BC3	TE	VIR	Jul	47	10	2/10 (20.0)	
BC3	TT	Water	Jul	42	10	1/10 (10.0)	3/39 (7.7)^c^
BC3	TT	BIO	May	45	10	0 /10	
BC3	TT	SYN	Jul	38	9	2/9 (22.2)	
BC3	TT	VIR	May	46	10	0/10	
Total				1567	344	82/344 (23.8)	

*BIO, Biosentry (Bisentry, Inc., Stone Mountain, GA, USA); SYN, Synergize (Preserve International, Reno, NV, USA; VIR, VirkonS (Dupont Animal Health Solutions, Sudbury, UK). †Superscript letters indicate significance, i.e., values between farms labeled with different letters differed significantly, but values between farms labeled with the same letter did not differ significantly. p<0.05 by binomial proportion test.

All 9 swine farms were positive for St-Valerien–like viruses. Overall prevalence was 23.8% (range 2.6%–80.0%) (Table 1). The prevalence in farm WL (32/40; 80.0%) was significantly higher than that in the other 8 farms, suggesting that an outbreak occurred at this farm during sampling. The prevalence in production system BC1 (41/119; 34.5%) was significantly higher than that in production system BC2 (18/107; 16.8%), but not BC3 (23/118; 19.5%). Differences among the 3 production systems were determined to be primarily due to the origin of the pigs. Each production system is fully independent with their own genetics/breeding units, farrowing sites where the sampled pigs originated, etc. Our findings suggest that breed differences or incidence of infection in pigs at earlier production stages might affect incidence at the sampled finisher stage. Overall, we found no statistically significant difference in prevalence among the treatments (biocides and water control) (data not shown). These results are consistent with the environmental stability of caliciviruses and their resistance to many disinfectants (*1*).

We also tested by RT-qPCR historical RNA samples extracted from pig fecal samples collected from December 2002 to March 2005 from 2 North Carolina farms, 4 Ohio farms, and 1 Michigan farm (n = 375) (Table 2). These RNA samples were from a previous study of the prevalence of porcine noroviruses and sapoviruses and have been stored at –70°C since 2005 (*12*). St-Valerien–like viruses were detected in 1 North Carolina swine farm in 2003 (10/19 samples; 53%). Only 1 of 60 samples collected in 2002 from the Michigan farm showed a weak positive result (3.3 × 10^5^ GE/g). No St-Valerien–like viruses were detected in the pigs of different ages on the 4 Ohio farms. These results suggest regional differences in the distribution of this new virus.

**Table 2:** Prevalence of St-Valerien–like viruses from historical pig fecal samples collected during 2002–2005, United States*

Swine farm	% Pigs (no. positive/total no.)				
	% Nursing pigs, 1–3 wk	% Post-weaning pigs, 3–10 wk	% Finisher pigs, 10–24 wk	% Sows, >1 y	% Total
Ohio A	0 (0/14)	0 (0/12)	0 (0/22)	0 (0/13)	0 (0/61)
Ohio B	0 (0/31)	0 (0/45)	0 (0/45)	0 (0/30)	0 (0/151)
Ohio C	0 (0/15)	0 (0/12)	0 (0/6)	0 (0/28)	0 (0/61)
Ohio D	0 (0/8)	0 (0/10)	NA	NA	0 (0/18)
North Carolina A	NA	NA	0 (0/5)	NA	0 (0/5)
North Carolina B	NA	NA	53 (10/19)	NA	53 (10/19)
Michigan A	NA	NA	2 (1/60)	NA	2 (1/60)
Total	0 (0/68)	0 (0/79)	7 (11/157)	0 (0/71)	3 (11/375)

*NA, not available.

**Figure 2:**
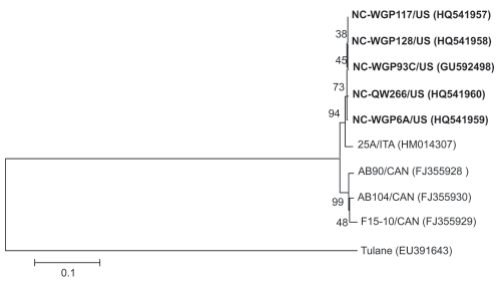
Neighbor-joining phylogenetic tree of St-Valerien–like viruses based on the predicted capsid viral protein 1 sequences (516 aa). The newly identified US St-Valerien–like virus strains are in **boldface**. The GenBank accession number of each strain is within parentheses. Bootstrap values are shown near branches. Rhesus monkey Tulane calicivirus was an outgroup control. Scale bar indicates amino acidsubstitutions per site.

Representative St-Valerien–like virus strains were selected on the basis of collection sites (Tables 1, 2): NC-WGP6A (BC2, FF, BIO), NC-WGP117 (BC1, WL, BIO), NC-WGP128 (BC1, WL, VIR), and 1 historic sample NC-QW266 (NC farm B). Because of low virus titers, the positive samples from production system BC3 and Michigan could not be further amplified. The 3′ end, 2,511-nt fragments, including the predicted partial RdRp, complete capsid viral protein (VP) 1, and minor structural protein VP2 genes were sequenced to examine the genetic variation among St-Valerien–like viruses. The 5 strains from the United States share 97.0%–99.9% nt identity in this region. Strains from the United States, the recently reported strain from Italy (25A), and the strains from Canada (*3**,**13*) share 96.4%–100%, 95.9%–100.0%, and 92.0%–100% aa identities for the partial RdRp (167 aa), VP1 and VP2, respectively. These results suggest that there is only 1 genotype within this potentially new genus (Figure 2), although the strain from Italy clusters with strains from the United States.

## Conclusions

St-Valerien–like viruses have been detected in Canada, the United States, and Italy. The prevalence of St-Valerien–like viruses in finisher pigs in North Carolina was 23.8%. No such viruses were detected in swine samples from Ohio collected during March 2003–March 2005. For a proposed new genus, it is critical to determine if St-Valerien–like viruses are present in other regions or species and to examine the genetic diversity among strains. Because these viruses are genetically closest to Tulane virus and human noroviruses (*2**,**3*), this information is useful in examining their potential for interspecies transmission and in controlling the spread of new viruses. Whether St-Valerien–like viruses cause disease in pigs or humans or cause food safety concerns requires further investigation.
